# Photocatalytic Degradation of Acetaminophen in Aqueous Environments: A Mini Review

**DOI:** 10.3390/toxics11070604

**Published:** 2023-07-12

**Authors:** Zhuowen Wang, Haijun Chen, Chang Rong, Anfeng Li, Xiuyi Hua, Deming Dong, Dapeng Liang, Haiyang Liu

**Affiliations:** Key Laboratory of Groundwater Resources and Environment, Ministry of Education, Jilin Provincial Key Laboratory of Water Resources and Environment, College of New Energy and Environment, Jilin University, Changchun 130012, China

**Keywords:** photocatalytic degradation, acetaminophen, photocatalyst, doping, heterojunction

## Abstract

Over the past few decades, acetaminophen (ACT), a typical nonsteroidal anti-inflammatory drug (NSAID), has gained global usage, positioning itself as one of the most extensively consumed medications. However, the incomplete metabolism of ACT leads to a substantial discharge into the environment, classifying it as an environmental contaminant with detrimental effects on non-target organisms. Various wastewater treatment technologies have been developed for ACT removal to mitigate its potential environmental risk. Particularly, photocatalytic technology has garnered significant attention as it exhibits high efficiency in oxidizing and degrading a wide range of organic pollutants. This comprehensive review aims to systematically examine and discuss the application of photocatalytic technology for the removal of ACT from aqueous environments. Additionally, the study provides a detailed overview of the limitations associated with the photocatalytic degradation of ACT in practical applications, along with effective strategies to address these challenges.

## 1. Introduction

**Figure 1:**
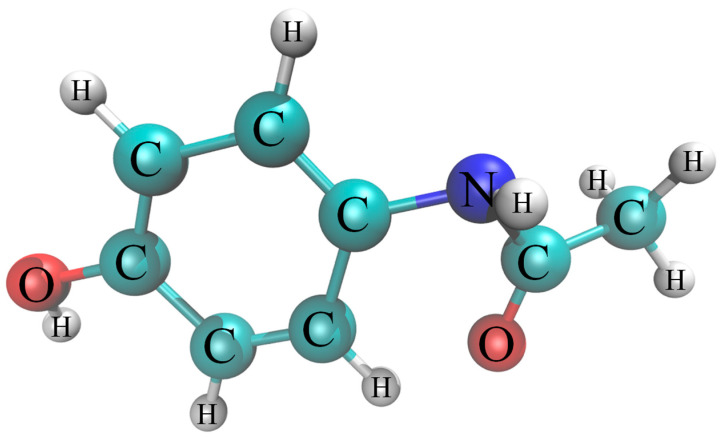
Chemical structure of ACT.

In recent decades, there has been a significant increase in the consumption of pharmaceuticals and personal care products (PPCPs) [1]. Unfortunately, due to poor absorption and incomplete metabolism, most PPCPs are discharged into the environment as their raw form or intermediates. These compounds are frequently detected in aqueous environments, leading to their emergence as contaminants due to their adverse effects [2]. Acetaminophen (ACT, N-(4-hydroxyphenyl) acetamide, Figure 1) is a commonly used therapeutic drug that is considered a highly persistent pollutant with uncertain and long-term effects on human health [3]. Despite a low ACT concentration, long-term exposure will cause a constant accumulation of toxicological effects, thereby inducing irreversible damage to human health and the ecosystem. For instance, physiological and morphological alterations in zebrafish have been widely reported in recent years [4,5]. Endocrine disruption and chronic diseases (including gastrointestinal, cardiovascular, and kidney diseases) may be also caused in humans by denaturing the proteins, damaging the genetic code, and oxidizing the lipids due to long-term exposure even at trace ACT concentration [6]. The hazardous and toxic property of these contaminants poses a serious threat to organisms and the ecosystem. Therefore, developing effective wastewater treatment technologies to remove these pollutants in aqueous environments is necessary. While traditional wastewater treatment technologies play a crucial role in controlling water pollution, they are flawed in several limitations, including high energy consumption, low treatment efficiency, and the potential for secondary pollution [7,8]. Consequently, it is crucial to develop wastewater treatment technologies that exhibit low energy consumption, high removal efficiency, and wide applicability to effectively address these issues.

As for ACT and its metabolites, extensive research has been carried out to address their removal from wastewater [9]. However, it is reported that conventional wastewater treatment technologies, including activated sludge, biodegradation, ozonation, and filtration, in wastewater treatment plants (WWTPs) are still far from satisfactory concerning the removal of ACT [10,11,12]. For instance, multiple bacterial strains were isolated from pharmaceutical effluents and used for the biodegradation of ACT, and the highest degradation efficiency was 92.35% by *Pseudomonas* strain PrS10 after 7 d treatment [13]. The most efficient technologies developed for this purpose are the advanced oxidation processes (AOPs), which are environmentally friendly methods relying on the oxidative degradation of organic pollutants by generating reactive oxygen species (ROS) [14]. Nevertheless, when the pollutants are diluted in large quantities, the application of typical AOPs (including electrocatalysis, Fenton, and ozonation) will become technically and economically difficult. Among various AOPs, photocatalytic advanced oxidation technology has overcome the usual rejection of AOPs and has gained significant attention in removing ACT due to its excellent degradation efficiency, low cost, and the potential for the solar-induced degradation of refractory pollutants [15,16,17].

In this review, along with discussing the adverse effects of ACT on human health and the ecosystem, the photocatalytic advanced oxidation technology for effective removal of ACT from aqueous solutions by different kinds of photocatalysts is comprehensively introduced. This study is organized into four sections to provide a comprehensive analysis. The first section presents essential information on the property and consumption of ACT. The second section examines the occurrence of ACT in various aqueous environments, including surface water, groundwater, and wastewater, followed by an overview of the potential impact of ACT on the ecosystem. In the third section, the focus is on the removal of ACT using photocatalytic AOPs. Specifically, it investigates the effectiveness of different photocatalysts in eliminating ACT from aqueous environments, including metal and nonmetal doping, co-doping photocatalysts, type-II heterojunction, and Z-scheme heterojunction photocatalysts. Finally, the fourth section discusses the primary challenge and future trends of applying photocatalytic AOPs in practical ACT treatment. This review provides a comprehensive overview of the photocatalytic degradation of ACT in aqueous environments by different photocatalysts.

## 2. ACT Contamination

### 2.1. ACT Properties and Usage

ACT, a representative nonsteroidal anti-inflammatory drug (NSAID), was first synthesized in the mid-19th century. It is also commonly known as paracetamol and has been widely used as a readily available over-the-counter (OTC) antipyretic and analgesic medication for treating fever, headache, and postoperative pain [18,19,20]. The pharmacological mechanism of ACT involves selectively inhibiting the synthesis of prostaglandins in the hypothalamic thermoregulatory center, helping regulate body temperature, and raising the pain threshold [21,22]. The detailed physicochemical properties of ACT are listed in Table 1.

ACT has gained extensive usage due to its significant therapeutic effects and low production cost, with annual consumption ranging from 4–50 tons per million inhabitants [25]. Notably, in the year 2000, the UK and Germany consumed 391 and 642 tons of ACT, respectively [26,27]. In 2003, South Korea produced 1069 tons of ACT [28], which was also the best-selling drug in France in 2010 [29]. In 2014, the consumption of ACT exceeded 149,300 tons globally [30]. However, absorption and full metabolization of ACT by the digestive system of organisms after ingestion is difficult. It was reported appropriately 58–68% of ACT had been released into the environment as prodrugs or intermediates during therapeutic use [31], and ACT removal is limited in conventional wastewater treatment plants (WWTPs) even at a trace concentration (100 μg/L) for a long treatment time (5 days) [32]. Consequently, ACT is frequently detected in various aqueous environments due to incomplete metabolism and degradation, thereby posing serious threats to ecological safety and human health [33].

### 2.2. Occurrences of ACT in Various Aqueous Environments

ACT contamination has emerged as a global environmental issue, with it widely detected in various aqueous environments, including surface water, groundwater, and municipal wastewater [34,35,36]. For instance, a median concentration of 0.11 μg/L ACT was detected in the US streams, and the concentration reached up to 6–10 µg/L in natural water [37]. The various residual drug, including ACT, has been detected in River Tyne with concentrations ranging from 4 to 10,000 ng/L [38]. Between 1999 and 2018, Peña-Guzmán et al. investigated the ACT concentration of 11 countries in Latin America; it was found that the ACT concentrations in wastewater and surface water ranged from 17.1–29,200 and 3–25,200 ng/L, respectively [39]. In California, the ACT concentration was up to 1.9 µg/L in groundwater [40], and in Korea, which increased nearly 11-fold from 6.8 to 75.0 µg/L in municipal wastewater from 2008 to 2012 [41,42]. Particularly, the residual ACT in the WWTPs effluent needs more attention (Table 2). It is reported that ACT has been widely detected from municipal WWTPs effluent in different countries, including Saudi Arabia (31,200 ng/L), the UK (6040 ng/L), United Arab Emirates (5235 ng/L), South Africa (3223 ng/L), and Canada (1932 ng/L) [43]. Additionally, the average ACT level in the effluent of Spanish WWTPs is reported to be 0.22 µg/L, which is approximately 6.7 times higher than that in the surface water (0.033 µg/L) [44]. Especially in developing countries, the residual pharmaceuticals from industrial effluent, municipal wastewater, hospital wastewater, and septic tank effluent are not collected and treated properly, resulting in a prevalence of ACT in natural aqueous environments [45]. Consequently, studies related to ACT degradation are urgently necessary to prevent its continuous accumulation in the natural aqueous environment and decrease its adverse effects on human health and ecosystems.

### 2.3. Adverse Effects of ACT

Despite the rapid effectiveness and apparent therapeutic benefits of ACT, the potential adverse effects during medication have often been overlooked. However, both domestic and international studies indicate that its prolonged use or overdose can result in serious health issues, such as fatal hepatotoxicity, liver disease, and pancreatic inflammation [51,52,53]. In the US alone, over 100,000 patients are hospitalized annually due to ACT overdose, with roughly 50% of these cases leading to liver failure [54]. Najafizadeh et al. reported that ACT overdose in humans and animals can cause uremia and renal tubular damage, with renal injury observed in at least 1–2% of patients [51]. ACT is listed as the fifth most common compound by the Environment Agency of England and Wales due to its prevalence and potential environmental risk [55]. Furthermore, the increasing ACT accumulation in aqueous environments may pose a serious threat to aquatic organisms. Studies have shown that ACT can lead to dysfunctional reproductive and endocrine systems in fish, which may also cause oxidative stress on other aquatic organisms, such as planktonic crustaceans and rotifers [56,57]. Kim et al. studied the acute toxicity of ACT using *Daphnia magna*, *V. fischeri*, and *O. latipes* as test organisms and found that semi-lethal concentrations (EC_50_) of ACT were 30.1, 549.7, and 160.0 mg/L, respectively. The calculated risk factor for ACT was 1.8, indicating its potential negative impact on the environment [58]. Additionally, ACT can interfere with the embryonic development, survival, and endocrine systems of aquatic organisms [59,60]. For instance, Cedron et al. investigated the acute toxicity of ACT using zebrafish embryos [4]. All zebrafish embryos incubating with 13.4 mM ACT died at 96 h, and only 5% of those exposed to 9.6 mM ACT survived by the end of the experiment. The test also revealed reduced pigmentation, impaired melanin synthesis, craniofacial structural abnormalities, pericardial edema, and blood accumulation in zebrafish embryos, indicating ACT could pose a possible teratogenic effect.

In conclusion, the presence of residual ACT in diverse aqueous environments poses a significant threat to both human health and aquatic organisms. As a result, the development of wastewater treatment technologies that can effectively degrade ACT and mitigate associated health risks is necessary.

## 3. Current Technologies for ACT Removal

Presently, wastewater treatment technologies can be broadly categorized into three groups: physical, biological, and chemical methods [1]. Physical methods, which mainly rely on adsorption techniques, have garnered significant attention due to their simplicity and cost-effectiveness. For instance, SBA-15 zeolite was synthesized and used as an adsorbent to remove ACT (4.4 ng/L) from wastewater, with a removal rate of about 92% [61]. Natarajan et al. designed mesoporous silica microspheres (MSMs) to adsorb ACT from the aqueous environment under the optimal conditions (pH 5, 1 g/L MSMs, 150 mg/L ACT); the ACT removal efficiency achieved 95.4% within 30 min [62]. Additionally, rhamno-lipid-coated cMNP (Rh-cMNP) was synthesized and used for the adsorption of ACT, and the removal efficiency of ACT (60 mg/L) could be 94.6% within 60 min [63]. However, the physical methods only achieve the temporary transfer of pollutants and cannot degrade organic pollutants completely.

In general, biological technologies can be classified into aerobic and anaerobic treatment methods [64]. For example, 400, 2500, and 2000 mg/L ACT could be completely removed by the aerobic method using the genera *Stenotrophomonas* sp. f1, *Pseudomonas* sp. F, and *Pseudomonas* sp. fg-2 within 116, 70, and 45 h, respectively [65]. Yang et al. evaluated the biodegradation of ACT in mangrove sediments, ACT-adapted sediment was supplemented with enzyme-containing microcapsules, and the degradation efficiency of ACT (2 mg/L) achieved 100.0% after 12 d under both aerobic and anaerobic conditions [66]. Although biological technologies can effectively remove ACT from the aqueous environment, they are limited in practical wastewater treatment due to their harsh operating conditions and slow degradation kinetics [67].

Furthermore, chemical methods encompass conventional chemical technology and AOPs, such as electrocatalytic oxidation, photocatalytic oxidation, and ozonation [1,68,69]. For instance, a reactive electrochemical membrane was prepared through carbon thermal reduction and used for the degradation of ACT. After 60 min of electrochemical reaction, ACT (6.7 µg/L) was completely removed at 15 mA/cm^2^ current density [70]. However, the practical application of electrochemical oxidation is limited in wastewater treatment due to its high-energy consumption and associated high treatment costs. As for ozonation, Mohebali et al. synthesized a novel Fe_3_O_4_@Ce-UiO-66 composite to catalytic ozonation for the degradation of ACT (25 mg/L), and only 14.1% total organic carbon (TOC) was reduced by a single ozonation process within 10 min, while 90.2% TOC could be removed in the presence of Fe_3_O_4_@Ce-UiO-66 [69]. It has also been reported that ozonation alone yielded unsatisfactory removal of 1 mol/L ACT, and the TOC removal efficiency was only 30% after 120 min [71]. Additionally, Fenton-like oxidation is capable of the efficient degradation of ACT from the aqueous environment. For instance, Tian et al. reported the degradation of ACT by Fenton-like oxidation under the optimal condition; the synthesized 5.5SACu-hsCN nanocomposite showed 94.8% of ACT degradation within 180 min [72]. However, Fenton-like technology is limited in practical application due to its high cost, strong acid condition (pH 3–4), and production of ferric sludge. Furthermore, the energy requirements for different AOPs are listed in Table 3. Considering the potential adverse effects of ACT on organisms at even extremely low concentrations and the limited removal efficiency of conventional wastewater treatment technologies, powerful and effective techniques are urgent to develop [28].

## 4. Photocatalytic Degradation of ACT

In recent years, photocatalytic AOPs have gained widespread application in the fields of energy and environment due to their eco-friendly property and cost-effectiveness [79,80]. The remarkable photocatalytic performance is primarily attributed to the excitation of electrons (e^−^) from the valence band (VB) to the conduction band (CB) upon exposure to sunlight, resulting in the accumulation of positively charged holes (h^+^) in the VB and negatively charged e^−^ in the CB [81]. Notably, the h^+^ generated in the VB can cleave water molecules, producing highly oxidizing •OH species [82]. Simultaneously, the e^−^ can be captured by oxygen molecules and gathered in the CB to generate •O_2_^−^, and more ROS can be generated as shown in Equations (1)–(4) [83,84]. The photocatalytic degradation process involves physical–chemical reactions on the surface of photocatalysts [85]. However, the rapid combination of the photogenerated e^−^–h^+^ pairs in photocatalysts results in energy loss, curtailing photocatalytic activity ultimately [86]. Therefore, controlling the kinetics of carrier processes to minimize the recombination of electron-hole pairs is crucial, as it facilitates the transfer of carriers to the photocatalyst surface. Various approaches have been proposed to modify the photocatalysts, aiming to enhance their photocatalytic degradation efficiency and chemical stability [87,88]. Currently, common modification strategies of photocatalysts mainly include doping and constructing heterojunctions.

O_2_ + e^−^ → •O_2_^−^ (1)

•O_2_^−^ + H^+^ → •HO_2_ (2)

•OOH → O_2_ + H_2_O_2_ (3)

H_2_O_2_ + e^−^ → •OH + OH^−^ (4)

### 4.1. Doping

In recent decades, the incorporation of metals and non-metals into photocatalysts through doping has attracted widespread attention since it can potentially enhance photocatalytic activity [89]. Doping enables improved utilization of visible light by creating lattice defects on the photocatalyst’s surface, which facilitates the trapping of photogenerated e^−^ [90]. The recombination of photogenerated e^−^–h^+^ pairs in photocatalysts can also be suppressed by doping [91]. Furthermore, doping with metals and non-metals is frequently employed to form various structures with unique morphologies and significant crystallinity in photocatalysts.

For instance, a Pd-BiVO_4_ photocatalyst was successfully synthesized by doping precious metal Pd nanoparticles on the BiVO_4_ surface [92]. The introduction of Pd broadened the absorption range of Pd-BiVO_4_ to 550 nm, and the Pd-BiVO_4_ nanocomposite exhibited excellent photocatalytic degradation performance, achieving the complete removal of ACT within 60 min of visible light irradiation with accompanied by a 40% mineralization rate. The degradation mechanism revealed that the generated h^+^ and •O_2_^−^ played major roles in ACT degradation in the Pd-BiVO_4_ photocatalytic system. Similarly, Ag-ZnO was successfully synthesized and achieved photocatalytic degradation efficiency of 90.8% for ACT within 120 min [93]. The photocatalytic property of ZnO was significantly improved after Ag-doping, with the rate constant being four times higher than that of pure ZnO. The enhanced charge transfer capability in the Ag-ZnO nanocomposite contributed to the improved degradation efficiency. Furthermore, photocatalytic degradation mechanism experiments showed that •OH played a major role in ACT removal. The Ag-ZnO photocatalyst maintained a removal rate of 75% even after five cycling experiments, indicating its efficiency and stability under visible light radiation. Additionally, a nano-sized Sb-TiO_2_ photocatalyst was also synthesized by doping non-precious metal Sb into TiO_2_ through a polymer precursor method [94]. The Sb-doped TiO_2_ photocatalyst exhibited reduced grain size and increased specific surface area with increasing Sb-doping dosage. The photocatalytic activity experiments showed that the photocatalytic degradation efficiency of the Sb-doped TiO_2_ photocatalyst was 1.5 times higher than that of the undoped TiO_2_.

Additionally, non-metal elements nitrogen (N), carbon (C), and boron (B) have been widely studied as dopants in recent years [95,96,97]. For example, N-doped ZnO (N-ZnO) was successfully prepared, which exhibited significant visible light absorption due to the Zn-N bonds and defects [98]. The degradation efficiency of ACT reached 98.5% after 120 min of photocatalytic reaction. The photogenerated e^−^–h^+^ pairs were effectively separated by N-doping, and the photocatalytic degradation activity was improved. Additionally, the degradation efficiency showed no obvious decrease after five recycling use, indicating that N-doping improved the stability of the photocatalyst significantly. The investigation of the degradation mechanism showed that •O_2_^−^ was the main ROS in the ACT degradation. C-doped graphitic carbon nitride (g-C_3_N_4_) was successfully synthesized with the advantages of high visible light utilization, strong VB h^+^ oxidation driving force, and effective separation of the photogenerated e^−^–h^+^ pairs [99]. Under visible light irradiation, multiple ROS, including •O_2_^−^, ^1^O_2_, and •OH, were generated and participated in the ACT degradation, significantly enhancing the degradation efficiency. B-doped TiO_2_ (B-TiO_2_) was successfully prepared with a small crystal size (25.62 nm) and a large specific surface area (17.23 m^2^/g) [100]. The results showed that the ACT (10 mg/L) removal efficiency could reach 98.8% by 4%B-TiO_2_ photocatalyst (1 g/L) after 30 min of ultraviolet (UV) light irradiation. Compared with pristine TiO_2_, B-doping optimized the structure and properties of photocatalysts due to more •OH could be generated in situ during the photocatalytic degradation of ACT. The radical scavenging experiments and electron spin resonance (ESR) results showed that •OH played a dominant role in the ACT degradation.

**Figure 2:**
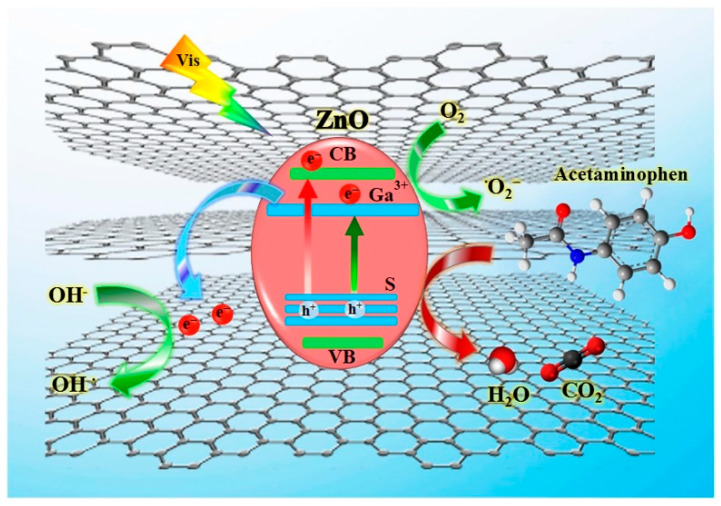
Mechanism of GaS@ZG as a visible-light-driven photocatalyst (Reprinted (adapted) with permission from [102]. Copyright 2020 Elsevier).

Co-doping, which involves the simultaneous doping of different types of atoms into a photocatalyst, has been recognized for its ability to enhance catalytic performance compared to undoped or single-atom-doped catalysts [101]. A novel type of Ga, S co-doped ZnO@rGO photocatalyst (GaS@ZG) was successfully prepared with excellent photocatalytic activity [102]. After 60 min of photocatalytic reaction, 50 mg/L ACT was completely degraded using the Ga_1.0_S_0.5_@ZG photocatalyst, and a 61.0% mineralization rate was reached. Compared with pure ZnO, the degradation efficiency of ACT increased eight times by the Ga_1.0_S_0.5_@ZG photocatalyst under optimal conditions. The GaS@ZG catalyst had the beneficial synergistic effects of expanding the solar spectrum utilization and promoting charge transfer. As shown in the degradation mechanism (Figure 2), the Ga, S co-doping reduced the bandgap energy and inhibited the photogenerated electron-hole combination, increasing charge utilization efficiency. It provided a novel co-doping photocatalyst for visible-light-driven photocatalytic degradation of pharmaceutical pollutants.

Paragas et al. developed a successful approach to preparing a visible-light-driven CeO_2_/IK-C_3_N_4_ photocatalyst using a simple thermal decomposition method [103]. Compared with pristine C_3_N_4_, the CeO_2_/IK-C_3_N_4_ nanocomposite showed a better photocatalytic performance with tunable optical properties and a suitable bandgap width, which was attributed to the fast charge separation by narrowing the bandgap. CeO_2_/IK-C_3_N_4_ exhibited excellent photocatalytic activity for ACT degradation, and the removal efficiency of ACT could reach 99.0% after 90 min of photocatalytic degradation. The performances of different metal and non-metal-doped photocatalysts for ACT degradation are summarized in Table 4. Hence, doping is an effective strategy for improving the photocatalytic performance of photocatalysts. The advantages of doping are mainly attributed to reducing bandgap and enhancing the separation efficiency of photogenerated e^−^–h^+^ pairs [104].

### 4.2. Heterojunction

The heterojunction refers to the interface between two semiconductor layers with different bandgaps and lattice constants [113]. In general, the heterojunctions mainly include conventional type-II and Z-scheme heterojunctions [114].

#### 4.2.1. Conventional Type-II Heterojunction

**Figure 3:**
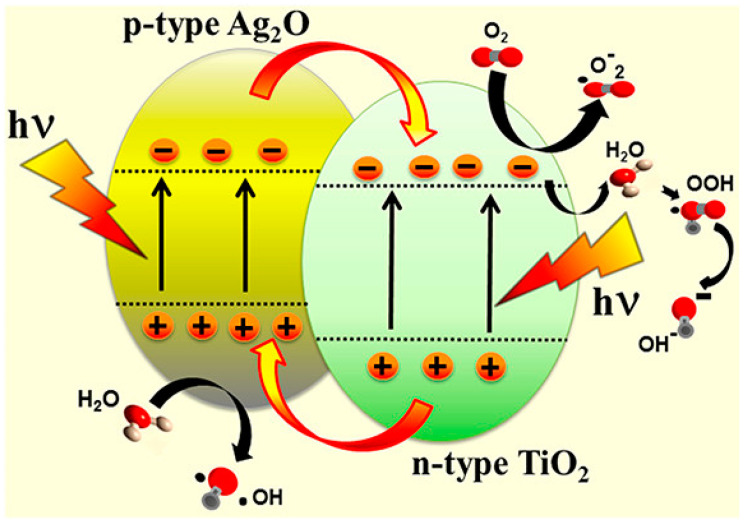
Electron transfer mechanism diagram in type-II heterojunction photocatalyst (Reprinted (adapted) with permission from [117]. Copyright 2013 American Chemical Society).

In a conventional type-II heterojunction (Figure 3), semiconductor I has a higher CB than semiconductor II. Under sunlight irradiation, e^−^ is transferred from semiconductor I to semiconductor II, while h^+^ is transferred from semiconductor II to semiconductor I, achieving effective separation of the photogenerated e^−^–h^+^ pairs [17]. Type-II heterojunctions are widely used in the photocatalytic degradation of ACT and other organic pollutants due to their fast charge transfer rate [115,116].

For instance, Khavar et al. successfully synthesized an Ag_2_S-ZnO@rGO Type-II heterojunction photocatalyst, exhibiting a smaller bandgap than pure ZnO and enhanced optical absorption properties [118]. Compared with pure ZnO and ZnO@rGO photocatalysts, the Ag_2_S-ZnO@rGO photocatalyst demonstrated the highest photocatalytic activity, achieving the complete degradation of 20 mg/L ACT in 60 min. The improved photocatalytic performance of the Ag_2_S-ZnO@rGO photocatalyst was attributed to the formation of a type-II heterojunction which effectively separated the photogenerated e^−^–h^+^ pairs. The radical scavenging experiments indicated that h^+^ and •O_2_^−^ were the main ROS in the photocatalytic degradation of ACT.

Peñas-Garzón et al. [119] successfully synthesized the TiO_2_-AZA4 photocatalyst, where AZA4 significantly reduced the bandgap energy of TiO_2_ and shifted its absorption edge toward the visible light range. Under visible light illumination, the as-synthesized TiO_2_-AZA4 nanocomposites demonstrated significantly enhanced photocatalytic activity. After 120 min of photocatalytic reaction, 5 mg/L ACT was completely photocatalytic degraded, and the degradation rate of the TiO_2_-AZA4 nanocomposites was three-fold higher than that of pristine TiO_2_. This was attributed mainly to the formation of type-II heterojunction with a narrow bandgap in the TiO_2_-AZA4 nanocomposites, resulting in the effective separation of photogenerated e^−^–h^+^ pairs. The •O_2_^−^ contribution was particularly significant during the photocatalytic degradation of ACT by TiO_2_-AZA4 nanocomposite. The synthesized TiO_2_-AZA4 showed good stability during recycling experiments, with only a slight decrease in degradation efficiency, highlighting its excellent chemical stability in treating contaminated water.

In addition, g-C_3_N_4_/UiO-66-NH_2_ photocatalyst, a highly efficient type-II heterojunction, was synthesized by incorporation of g-C_3_N_4_ with MOF UiO-66-NH_2_ [120]. After 240 min of photocatalytic reaction, 5 mg/L ACT could be completely degraded by g-C_3_N_4_/UiO-66-NH_2_ photocatalyst. Moreover, g-C_3_N_4_/UiO-66-NH_2_ showed good stability and reusability, with only a slight decrease in photocatalytic degradation efficiency in the recycling experiments. During the photocatalytic degradation process, h^+^ and •O_2_^−^ were identified as the primary ROS. The performances of different type-II heterojunction photocatalysts in ACT degradation are summarized in Table 5.

Despite the high efficiency of type-II heterojunction photocatalysts in separating photogenerated e^−^–h^+^ pairs, several challenges impede their practical application. One obstacle is the occurrence of reduction reactions at low potentials within the semiconductors, which diminishes the redox capability of type-II heterojunction photocatalysts [125]. Additionally, the low oxidation-reduction potential in type-II heterojunction photocatalysts hampers the generation of ROS for the photocatalytic degradation of target pollutants [126]. Therefore, it is imperative to develop more effective heterostructure photocatalysts to enhance their photocatalytic performance.

#### 4.2.2. Z-Scheme Heterojunction

**Figure 4:**
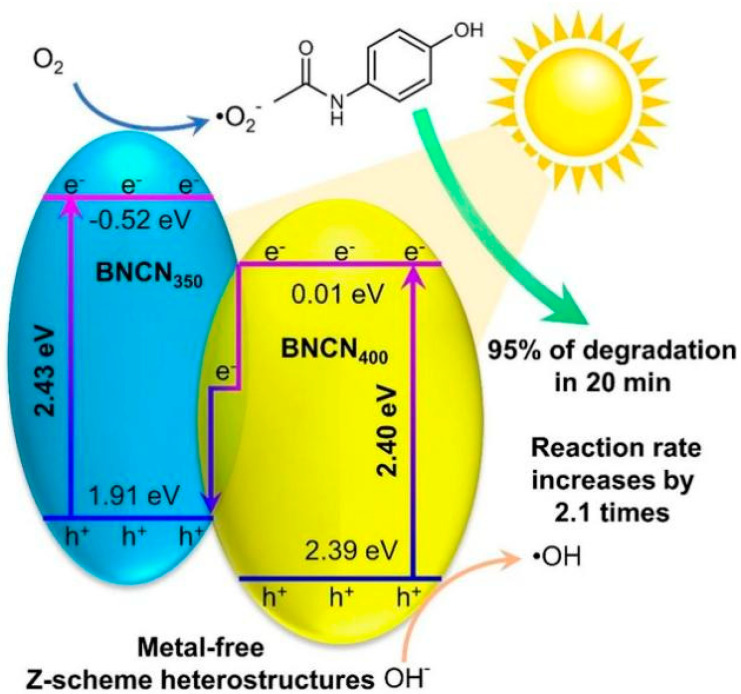
Schematic illustration of the photocatalytic degradation of ACT by BNCN_350_/BNCN_400_ Z-scheme heterojunction photocatalyst (Reprinted (adapted) with permission from [127]. Copyright 2023 Elsevier).

The concept of the Z-scheme heterojunction has been proposed as a solution to address these challenges. In a Z-scheme photocatalyst, the recombination process takes place between e^−^ in the CB with a smaller negative potential in one semiconductor and h^+^ in the VB with a smaller positive potential in another semiconductor (Figure 4) [127]. As a result, the overall oxidation-reduction potential of the Z-scheme catalyst system is increased. The Z-scheme system effectively utilizes excess e^−^ and h^+^ to maintain a high oxidation-reduction potential of the catalytic system, thereby enhancing the photocatalytic activity [128].

For instance, BNCN_350_/BNCN_400_ (a boron-doped nitrogen-deficient C_3_N_4_) Z-scheme heterostructure photocatalyst was successfully prepared and applied to the rapid photocatalytic degradation of ACT [127]. Under simulated sunlight irradiation, the removal efficiency of ACT was close to 100% within 30 min. This excellent photocatalytic performance is attributed to the fact that the BNCN_350_/BNCN_400_ Z-scheme heterostructure enhances the light absorption range and the separation efficiency of photogenerated e^−^–h^+^ pairs. Therefore, assembling N-C into a Z-scheme heterojunction can significantly improve photocatalytic degradation efficiency without using any metal-based materials. In addition, Moradi et al. successfully prepared a TiO_2_/graphene/g-C_3_N_4_ Z-scheme heterojunction photocatalyst (TGCN), significantly improving the photocatalytic degradation efficiency of ACT under simulated solar light irradiation [129]. Under the optimal condition, ACT (50 mg/L) was completely degraded by TGCN nanocomposite after 120 min of photocatalytic reaction. The results showed that the improved photocatalytic performance of TGCN was mainly due to the enhanced separation of photogenerated e^−^–h^+^ pairs and promoted charge transfer on the interface, generating a large amount of •OH and •O_2_^−^. Additionally, the degradation efficiency of ACT by TGCN only slightly decreased to about 95.8% after six consecutive cycles, indicating the good stability and reusability of the prepared TGCN nanocomposite. The radical scavenging experiments showed that •OH and •O_2_^−^ played a dominant role in ACT degradation. Therefore, TGCN exhibited excellent photocatalytic performance for removing refractory pollutants such as ACT under simulated solar light irradiation.

Liu et al. synthesized a TiO_2_/C-deficient g-C_3_N_4_ Z-scheme heterojunction (TiO_2_/VC-CN) photocatalyst by constructing a Ti-N coordination bridge, which significantly improved the photogenerated carrier separation efficiency [130]. Using TiO_2_/VC-CN, 10 mg/L ACT was completely removed in 90 min. The Ti-N coordination bond formed at the interface of TiO_2_ and VC-CN, which plays an important role in the charge transfer channel by shortening the carrier transfer distance. Many ROS, such as •O_2_^−^, •OH, and ^1^O_2_, were generated in the TiO_2_/VC-CN system and promoted the photocatalytic oxidation performance of TiO_2_/VC-CN for removing ACT from water. In addition, the O-doped WO_3_/g-C_3_N_4_ Z-scheme heterojunction photocatalyst was synthesized using a hydrothermal method by in situ acid-induced self-assembly of melamine and sodium tungstate [131]. The photocatalytic degradation efficiency of ACT (10 mg/L) achieved 98.2% after 60 min of photocatalytic reaction. The photocatalyst maintained high catalytic activity even after five experimental cycles. The performances of different Z-scheme heterojunction photocatalysts for ACT degradation are summarized in Table 6. In summary, the Z-scheme heterojunction photocatalyst demonstrates high ACT photocatalytic degradation efficiency due to its large specific surface area, enhanced visible light absorption, and accelerated interfacial charge transfer and separation.

### 4.3. Photocatalytic Degradation Kinetics

For the photocatalytic degradation of ACT with different photocatalysts, the comparison of degradation kinetic rate is crucial. For instance, IL-Fe/TiO_2_(Ar) photocatalyst was synthesized and used for the photocatalytic degradation of ACT under UV irradiation. Under the optimal conditions, the ACT degradation efficiency was 90.35% within 90 min, and the rate constant of the pseudo-first-order was 0.25 min^−1^ [108]; additionally, one-dimensional N-TiO_2_ nanotubes were fabricated by atomic layer deposition and applied to degrade ACT under visible light irradiation. Under the optimal conditions, more than 98% of ACT was degraded by N-TiO_2_ nanotube photocatalyst within 180 min, and the rate constant was 0.045 min^−1^ [109]; Shaban et al. successfully prepared carbon-doped titanium oxide (CTiO_2_) nanocomposites by the sol-gel method, and almost 100% of ACT was photocatalytic degraded within 90 min. The kinetic results indicated that the photocatalytic degradation of ACT using CTiO_2_ photocatalyst followed a pseudo-first-order reaction kinetic with a degradation rate of 0.082 min^−1^ [111]. Furthermore, g-C_3_N_4_-CdS/Bi_4_O_5_I_2_ composite photocatalyst was synthesized with two charge transfer pathways, and the ACT degradation efficiency by g-C_3_N_4_-CdS/Bi_4_O_5_I_2_ composite under visible light irradiation achieved 80% after 25 min. The photocatalytic degradation process followed a pseudo-first-order model, and the rate constant was 0.063 min^−1^ [122]. Hence, the photocatalytic performance by different photocatalysts can be directly reflected by describing the photocatalytic degradation reaction rate.

### 4.4. Degradation Mechanism

**Figure 5:**
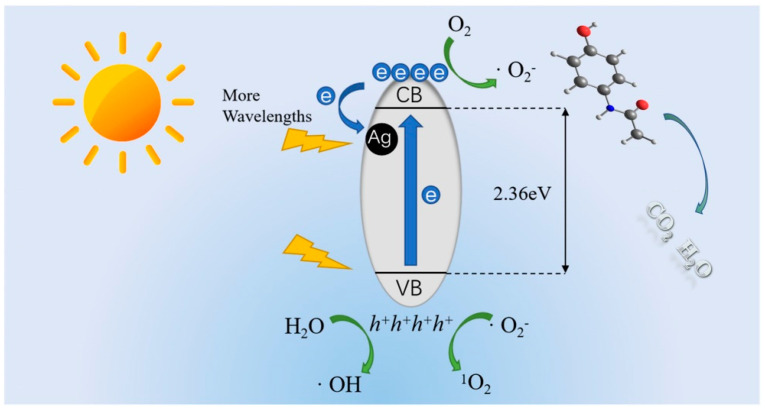
Illustration summarizing the proposed degradation mechanism by different ROS (Reprinted (adapted) with permission from [136]. Copyright 2023 Elsevier).

In general, the photocatalytic degradation of organic pollutants is achieved relying on the oxidative property of generated ROS, and the contribution of different ROS during the photocatalytic degradation process is always investigated by combining the results of scavenger experiments and electron spin resonance (ESR) spectroscopy. For instance, the contribution of different ROS was indirectly identified by the scavenger experiment and ESR technology during the photocatalytic degradation of ACT using single-atom Ag-loaded carbon nitride photocatalysts (Figure 5) [136]. In brief, adequate scavengers, including EDTA-2Na for h^+^, *tert*-butanol (TBA) for •OH, *p*-benzoquinone (PBQ) for •O_2_^−^, and furfuryl alcohol (FFA) for ^1^O_2_, were added into the photocatalytic degradation system, respectively. Additionally, the generation of ^1^O_2_, •OH, and •O_2_^−^ were further verified using the ESR technology in which the •OH and •O_2_^−^ were investigated using DMPO (5,5-dimethyl-1-pyrolin-N-oxide) as the trapping agent, and ^1^O_2_ was captured by TEMPO (2,2,6,6-tetramethyl-1-piperidinyloxy), respectively. Combing the results of the scavenger experiment and ESR analysis, it was found that •O_2_^−^ played the major contribution to ACT degradation, and ^1^O_2_ made a moderate contribution.

## 5. Challenges and Future Trends of Photocatalytic Degradation of ACT

Enhancing the catalytic activity of the photocatalysts is crucial for the development of a viable technology for ACT removal. Despite extensive research in this field over the years, there are still several challenges that limit its practical application. One major challenge is the reusability of photocatalysts. The photocatalyst in the photocatalytic degradation system is generally recovered by centrifugation [93]. Although centrifugal recovery is effective in laboratory settings, it can lead to a gradual loss of the photocatalyst material in practical applications. The photocatalyst nanocomposite can be lost gradually with the increased number of cycles. Therefore, the centrifugal recovery rate shows a downward trend [100]. In addition, centrifugal recovery has high technical demands, and the installation of large-scale centrifuges is needed in practical applications, which significantly adds to the economic costs [126].

Second, a crucial factor impacting the practical application of photocatalysts is their stability. For instance, the catalytic activity of the photocatalyst decreases when used continuously, which is caused by the adverse effects of pollutants and intermediates on the surface of the photocatalyst. The chemical stability of photocatalysts can be verified by recycling experiments. In addition, analyzing the Fourier transform infrared spectra (FT-IR) and X-ray diffraction (XRD) of the photocatalysts before and after the photocatalytic reaction are effective in investigating their chemical stability [103,105,120].

Achieving complete mineralization of ACT is also a significant challenge. Most studies have shown that the photocatalytic degradation of ACT ultimately generates numerous intermediates, resulting in a lower mineralization efficiency compared to the degradation efficiency [137,138,139]. Moreover, compared with the parent compound, the potential toxicity of generated intermediates might be increased, diminishing the practical significance of photocatalytic degradation [140].

Furthermore, the cost, energy efficiency, or overall operating cost of photocatalytic AOPs have often been overlooked in most studies [126]. However, the cost cannot be disregarded in practical applications, as it must meet operational requirements within a reasonable budget. Therefore, cost considerations pose a significant and crucial challenge that hinders the practical implementation of photocatalytic technology in ACT degradation.

## 6. Conclusions and Perspective

In summary, this study provides an overview of the application of advanced photocatalytic oxidation technology in the removal of ACT. Different kinds of photocatalysts, including metal and nonmetal doping, co-doping, type-II heterojunction, and Z-scheme heterojunction photocatalysts, are discussed for the photocatalytic degradation of ACT from the aqueous environment. Photocatalytic technology meets the needs of a sustainable society and has demonstrated exceptional performance in ACT degradation due to its unique advantages. However, further improvements are needed in terms of photocatalytic degradation efficiency, economic cost, reusability, and chemical stability of photocatalysts.

The comprehensive impact of ACT on the ecosystem is not fully understood despite continuous research on ACT degradation. The following standpoints are suggested: (1) Establishing comprehensive ACT emission standards is necessary. Issuing permissible emission standards and allowable concentration in the environment may assist researchers in conducting further studies; (2) strict regulations must be formulated and enforced regarding the discharge of pharmaceutical wastewater; (3) studying the bioaccumulation, migration, and biodegradability of ACT to monitor its potential impact on the ecological and environmental safety is necessary; (4) developing low-cost and highly-efficient photocatalysts with remarkable reusability and chemical stability is crucial; (5) further research for ACT degradation in actual wastewater using visible-light-driven photocatalysts is needed.

## Figures and Tables

**Table 1:** Physicochemical properties of ACT [23,24].

Properties	Values
Chemical formula	C_8_H_9_NO_2_
CAS number	103-90-2
Molecular weight (g/mol)	151.2
Melting point (°C)	168–172
Vapor pressure (mmHg)	5.2 × 10^−6^
Log *K*_ow_	2.0
Henry’s constant (at m^3^/m)	6.4 × 10^−13^
Water solubility (mg/L)	1400–2400

**Table 2:** ACT concentration in different WWTPs effluent.

Country	Concentration (ng/L)	Year	Ref.
Canada	320	2018	[46]
Colombia	25–410	2018	[47]
India	330–1200	2017	[48]
Spain	17–441	2018	[49]
Turkey	436	2019	[50]

**Table 3:** The energy requirements for different AOPs.

AOPs	Pollutant	Energy Requirements (kWh/m^3^/Order)	Ref.
Electrocatalysis	Crystal violet	0.7	[73]
Electrocatalysis	Bisphenol A	0.07	[74]
Electrocatalysis	Nitrobenzene	2.07	[75]
Photocatalysis	Phenol	38.9–47.1	[76]
Ozonation	Phenol	26.2	[76]
Ozonation	Humic- and fulvic-like organics	18.0	[77]
UV-activated persulfate	Furfural	20.9	[78]
UV-activated peroxide	Furfural	34.5	[78]
UV-activated percarbonate	Furfural	26.6	[78]

**Table 4:** Different photocatalysts with metal and non-metal doping for the photocatalytic degradation of ACT.

Catalyst	Type	Light Source	*C*_ACT_ (mg/L)	Time (min)	Efficiency (%)	Ref.
Pt/NH_2_-MIL-125	Pt-doped	Xe lamp(λ ≤ 290 nm filter)	5	180	100.0	[105]
Pd-BiVO_4_	Pd-doped	Xe lamp	10	60	100.0	[92]
Al-TiO_2_/Al_2_O_3_	Al-doped	UV lamp	40	300	85.0	[106]
Sb-TiO_2_	Sb-doped	UVA LED	4.6	120	70.0	[94]
Bi^3+^-TiO_2_	Bi^3+^-doped	UVA lamp	15	240	98.0	[107]
Fe/TiO_2_	Fe-doped	UVC lamp	10	90	91.4	[108]
Ag-ZnO	Ag-doped	Halogen lamp	5	120	90.8	[93]
B-TiO_2_	B-doped	Halogen lamp	10	30	98.8	[100]
N-TiO_2_ NTs	N-doped	Halogen lamp	5	90	98.3	[109]
HNT/TiO_2_	N-doped	Halogen lamp	10	270	95.0	[110]
N-ZnO	N-doped	Sunlight	20	120	98.5	[98]
C-TiO_2_	C-doped	UV lamp	3	90	100.0	[111]
HCN-C_x_	C-doped	Xe lamp	10	10	98.4	[99]
C-DCN	C-doped	Xe lamp	10	60	99.4	[112]
GaS@ZG	Ga, S co-doped	Xe lamp	50	60	100.0	[102]
CeO_2_/IK-C_3_N_4_	CeO/I, K-co-doped	Visible light	10	90	99.0	[103]

**Table 5:** Type-II heterojunction photocatalyst for the photocatalytic degradation of ACT.

Catalyst	Light Source	*C*_ACT_ (mg/L)	Time (min)	Efficiency (%)	Ref.
Cu_2_O/WO_3_/TiO_2_	Xe lamp	1	60	92.5	[121]
g-C_3_N_4_-CdS/Bi_4_O_5_I_2_	Xe lamp	3	25	80.0	[122]
TiO_2_-AZA4	Xe lamp (λ ≤ 320 nm filter)	5	120	100.0	[119]
Sr@TiO_2_/UiO-66-NH_2_-2	Xe lamp (λ ≤ 320 nm filter)	5	240	93.5	[17]
Ag_2_S-ZnO@rGO	Xe lamp	20	60	100.0	[118]
g-C_3_N_4_/UiO-66-NH_2_	UVA	5	240	100.0	[120]
TiO_2_/0.25Nb_2_O_5_	40 W LED	30	20	90.6	[123]
SnO_2_@ZnS	Mercury lamp	10	120	70.0	[124]

**Table 6:** Z-scheme heterojunction for the removal of acetaminophen.

Catalyst	Light Source	*C*_ACT_ (mg/L)	Time (min)	Efficiency (%)	Ref.
COF-PD/AgI	Visible light	5	160	100.0	[132]
BNCN_350_/BNCN_400_	Xe lamp	10	30	100.0	[127]
TiO_2_/VC-CN	Xe lamp	10	90	100.0	[130]
g-C_3_N_4_/TiO_2_	Xe lamp	10	60	99.0	[133]
WO_3_/g-C_3_N_4_	Xe lamp	10	60	98.2	[131]
TiO_2_/g-C_3_N_4_	Xe lamp	10	45	96.7	[134]
Bi_2_O_3_/rGO/Mo_n_O_3n-1_	Xe lamp	10	360	76.5	[135]
TiO_2_/graphene/g-C_3_N_4_	Xe-lamp (λ ≤ 420 nm filter)	50	120	100.0	[129]
